# Supplementary material for: Light-sheet photonic force optical coherence elastography for high-throughput quantitative 3D micromechanical imaging
Source: Nat Commun. 2022 Jun 16;13:3465. doi: 10.1038/s41467-022-30995-0 (PMC9203576; doi:10.1038/s41467-022-30995-0)
Supplement: Supplementary file 3 — Description of Additional Supplementary Files [file 41467_2022_30995_MOESM3_ESM.docx]

**Description of Additional Supplementary Files:**

**Supplementary Video 1:** Visualization of Fig. 3b from different angles.

**Supplementary Video 2:** Visualization of Fig. 3c from different angles.
