# Supplementary material for: Light-sheet photonic force optical coherence elastography for high-throughput quantitative 3D micromechanical imaging
Source: Nat Commun. 2022 Jun 16;13:3465. doi: 10.1038/s41467-022-30995-0 (PMC9203576; doi:10.1038/s41467-022-30995-0)
Supplement: Supplementary file 6 — Reporting Summary [file 41467_2022_30995_MOESM6_ESM.pdf]

nature portfolio

Corresponding author(s): Steven G. Adie

Last updated by author(s): Apr 29, 2022

Reporting Summary

Nature Portfolio wishes to improve the reproducibility of the work that we publish. This form provides structure for consistency and transparency in reporting. For further information on Nature Portfolio policies, see our Editorial Policies and the Editorial Policy Checklist.

Statistics

For all statistical analyses, confirm that the following items are present in the figure legend, table legend, main text, or Methods section.

n/a	Confirmed	
☐	☒	The exact sample size (*n*) for each experimental group/condition, given as a discrete number and unit of measurement
☐	☒	A statement on whether measurements were taken from distinct samples or whether the same sample was measured repeatedly
☐	☒	The statistical test(s) used AND whether they are one- or two-sided<br>*Only common tests should be described solely by name; describe more complex techniques in the Methods section.*
☒	☐	A description of all covariates tested
☒	☐	A description of any assumptions or corrections, such as tests of normality and adjustment for multiple comparisons
☐	☒	A full description of the statistical parameters including central tendency (e.g. means) or other basic estimates (e.g. regression coefficient) AND variation (e.g. standard deviation) or associated estimates of uncertainty (e.g. confidence intervals)
☐	☒	For null hypothesis testing, the test statistic (e.g. *F*, *t*, *r*) with confidence intervals, effect sizes, degrees of freedom and *P* value noted<br>*Give P values as exact values whenever suitable.*
☒	☐	For Bayesian analysis, information on the choice of priors and Markov chain Monte Carlo settings
☒	☐	For hierarchical and complex designs, identification of the appropriate level for tests and full reporting of outcomes
☐	☒	Estimates of effect sizes (e.g. Cohen's *d*, Pearson's *r*), indicating how they were calculated

Our web collection on statistics for biologists contains articles on many of the points above.

Software and code

Policy information about availability of computer code

Data collection	LabVIEW 2014 64-bit are used for data acquisition. CT-FIRE V2.0 Beta (Bredfeldt, Jeremy S., et al.) are used for microfibrous structures segmentation and analysis.
Data analysis	MATLAB 2017a are used to conduct OCT image reconstruction and primary OCE analysis. Python v3.7.7 are used for further analyzing and visualizing results. Custom codes for generating plots in the main manuscript are available at Harvard Dataverse (https://doi.org/10.7910/DVN/IJXI4P).

For manuscripts utilizing custom algorithms or software that are central to the research but not yet described in published literature, software must be made available to editors and reviewers. We strongly encourage code deposition in a community repository (e.g. GitHub). See the Nature Portfolio guidelines for submitting code & software for further information.

Data

Policy information about availability of data

All manuscripts must include a data availability statement. This statement should provide the following information, where applicable:

- Accession codes, unique identifiers, or web links for publicly available datasets
- A description of any restrictions on data availability
- For clinical datasets or third party data, please ensure that the statement adheres to our policy

The data used to produce the plots within this paper are available at Harvard Dataverse (https://doi.org/10.7910/DVN/IJXI4P). All other data used in this study are available from the corresponding authors upon reasonable request.

Field-specific reporting

Please select the one below that is the best fit for your research. If you are not sure, read the appropriate sections before making your selection.

☒ Life sciences ☐ Behavioural & social sciences ☐ Ecological, evolutionary & environmental sciences

For a reference copy of the document with all sections, see nature.com/documents/nr-reporting-summary-flat.pdf

Life sciences study design

All studies must disclose on these points even when the disclosure is negative.

Sample size	The size of cells used in main studies was between seven to ten and each cell was from different culturing petri-dish under same culturing conditions. The size of cells used in control studies was at least three, with same sample conditions to main studies. No statistic model was used to calculate the sample size. The sample size was determined by the requirement of statistic significance and prior experiences (Han, Yulong, et al, PNAS, 115(16), 2018).
Data exclusions	No data was excluded for all experiments.
Replication	All results reported were conducted in at least three different measurements independently, and the results were consistent.
Randomization	Cells that meet the criteria of elongated morphology and orientation (described in Methods) were selected for measurement at random. Specific locations of micromechanical measurements within each sample were statistically sampled by the random distribution of beads dispersed in the sample.
Blinding	Data processing and analysis was fully automated, with the same processing and analysis parameters applied across all datasets being compared.

Reporting for specific materials, systems and methods

We require information from authors about some types of materials, experimental systems and methods used in many studies. Here, indicate whether each material, system or method listed is relevant to your study. If you are not sure if a list item applies to your research, read the appropriate section before selecting a response.

Materials & experimental systems

n/a	Involved in the study	
☒	☐	Antibodies
☐	☒	Eukaryotic cell lines
☒	☐	Palaeontology and archaeology
☒	☐	Animals and other organisms
☒	☐	Human research participants
☒	☐	Clinical data
☒	☐	Dual use research of concern

Methods

n/a	Involved in the study	
☒	☐	ChIP-seq
☒	☐	Flow cytometry
☒	☐	MRI-based neuroimaging

Eukaryotic cell lines

Policy information about cell lines

Cell line source(s)	NIH-3T3 fibroblasts were sourced from ATCC (CRL-1658).
Authentication	Cell line used behaved as expected and reported in the literature but was not authenticated.
Mycoplasma contamination	Cell line was tested by vendor for mycoplasma contamination.
Commonly misidentified lines (See ICLAC register)	No commonly misidentified cell lines were used.
